# A comparison of immediate release and delayed release cysteamine in 17 patients with nephropathic cystinosis

**DOI:** 10.1186/s13023-021-01991-2

**Published:** 2021-09-14

**Authors:** Christina van Stein, Sabrina Klank, Marianne Grüneberg, Chris Ottolenghi, Jürgen Grebe, Janine Reunert, Erik Harms, Thorsten Marquardt

**Affiliations:** 1 Department of General Pediatrics, Metabolic Diseases, University of Muenster, Albert-Schweitzer-Campus 1, 48149 Muenster, Germany; 2 UMR 1163, Université Paris Descartes, Sorbonne Paris Cité, Institut IMAGINE, 24 Boulevard du Montparnasse, 75015 Paris, France; 3 Biochimie Métabolique et Protéomique, Hôpital Necker – Enfants Malades, 149 Rue de Sèvres, 75015 Paris, France

**Keywords:** Nephropathic cystinosis, Immediate-release cysteamine, Delayed-release cysteamine

## Abstract

**Background:**

Nephropathic cystinosis is a rare and severe metabolic disease leading to an accumulation of cystine in lysosomes which especially harms kidney function. A lifelong therapy with the aminothiol cysteamine can delay the development of end-stage renal disease and the necessity of kidney transplantation. The purpose of our study was to compare the effectiveness of immediate-release and delayed-release cysteamine on cystine and cysteamine levels as well as assessing the onset of adverse effects.

**Methods:**

We retrospectively analysed cystine and cysteamine levels of 17 patients after a single dose of immediate-release cysteamine (Cystagon®, Mylan Pharmaceuticals, Canonsburg, PA and Recordati Pharma GmbH) as well as a single dose of delayed-release cysteamine (Procysbi®; Horizon Pharma USA and Chiesi Farmaceutici S.p.A., Parma, Italy) respectively. Data were collected during a period of three years in the context of optimizing the individual treatment regimens. The dose of DR-cysteamine was reduced to 70% of the equivalent dose of IR-cysteamine. The efficacy of both formulas in depleting white blood cells’ cystine levels and their side effects were compared.

**Results:**

Immediate (IR)- and delayed-release (DR) cysteamine effectively decreased intracellular cystine levels under the target value of 0.5 nmol cystine/mg protein, while fewer side effects occurred under DR-cysteamine. Mean maximum levels of cysteamine were reached after 60 min with IR-cysteamine and after 180 min with DR-cysteamine.

**Conclusion:**

A therapy with DR-cysteamine is as effective as IR-cysteamine while less side effects were reported. Our data show that DR-cysteamine should be dosed higher than 70% of the equivalent dose of IR-cysteamine in order to decrease cystine levels over an extended period of time. Moreover, our data suggest increasing the dosing scheme of Procysbi® to three times daily, to prevent a rapid decrease and achieve a steadier decline in cystine levels. Due to the more convenient dosing scheme, DR-cysteamine might ameliorate therapy adherence and improve patients’ quality of life.

## Background

Nephropathic cystinosis is a lysosomal storage disease which is inherited in an autosomal-recessive manner. Due to its low estimated prevalence of 1:100,000–200,000 [1], it is considered an orphan disease. Different mutations in the CTNS gene lead to an impaired function of the transport protein cystinosin in the lysosomal membrane which removes cystine from the lysosomes [2]. Consequently, cystine accumulates in the lysosomes, harming various organs, primarily the kidneys [3].

The most common form of nephropathic cystinosis is the infantile one (95%). The main clinical symptom is Fanconi syndrome. It is characterized by a failure to thrive, rickets, electrolyte imbalances, polyuria and polydipsia and develops within in the first year of life [1]. Without treatment, end-stage renal disease occurs on average at the age of 9 years [4]. Further systemic complications are growth retardation, hypothyroidism, photophobia caused by corneal cystine crystals, diabetes mellitus and hypogonadism [1]. The two other less common allelic forms of nephropathic cystinosis are the ocular form with an involvement of the eyes only [5] and the intermediate form, which shows less severe clinical symptoms [6].

The diagnosis of nephropathic cystinosis consists of the typical clinical findings as described above, as well as the detection of elevated cystine levels in white blood cells (WBC), which are also used to monitor the effectiveness of the therapy. Additionally, a genetic analysis to determine the gene mutations is recommended.

As the development of end-stage renal disease is common in patients with nephropathic cystinosis, the advance of renal transplantation has greatly improved patients’ life expectancy.

Besides symptomatic therapy, specific therapy consists of a lifelong intake of the aminothiol cysteamine, which first was shown to reduce cystine levels in 1976 by Thoene et al. [7].

An immediate-release form of cysteamine bitartrate (Cystagon®; Mylan Pharmaceuticals, Canonsburg, PA and Recordati Pharma GmbH) was approved by the FDA in 1994 and by the EMA in 1997. Cysteamine is an aminothiol which enters the lysosomes and reacts with cystine. The resulting mixed disulfide cysteine-cysteamine and cysteine can exit the lysosome via cysteine and lysine carriers [8]. After the mixed disulfide is reduced to cysteamine and cysteine, cysteamine can reenter the lysosomes. Thus, the lysosomes are effectively cleared of cystine. Several studies have shown that a regular intake of cysteamine, started as early in life as possible, results in improved growth, a longer time until kidney transplantation and an improved life expectancy [9, 10]. However, due to its frequent dosing schedule of 4 times per day, a daily interruption of sleep is inevitable. Additionally, numerous side effects, such as nausea and sulfurous body odor (halitosis) are obstacles to therapy adherence, especially with adolescent patients [11, 12].

A formula of cysteamine with a delayed release (Procysbi®; Horizon Pharma USA and Chiesi Farmaceutici S.p.A., Parma, Italy) has been developed and was approved by the FDA and European Commission in 2013. DR-cysteamine is administered in the form of gelatin capsules containing pellets, that are resistant to gastric acid. DR-cysteamine is released in the small intestine, while IR-cysteamine is released in the stomach. Besides these two approved pharmaceutical forms of cysteamine, some patients are treated with a self-manufactured formula of cysteamine, which is encapsulated by a pharmacist by using polymers, likewise causing a delayed release [13].

Our study compared immediate-release cysteamine (Cystagon®) and delayed-release cysteamine (Procysbi®) in terms of their effectivity in decreasing white blood cell cystine content as well as the onset of side effects in 17 patients.

## Methods

Pharmacokinetic parameters as well as baseline characteristics of 17 patients with nephropathic cystinosis were analysed retrospectively.

### Data collection

Data had been collected during a period of three years in the context of a standard operating procedure: at the department of General Pediatrics at the University Hospital Muenster, patients with nephropathic cystinosis receive one-time measurements of their cysteamine and cystine levels with IR-cysteamine as well as DR-cysteamine, aiming to assess the optimal therapy for each patient, regarding effectiveness on white blood cells (WBC) cystine levels, as well as the occurrence of side effects.

The patient collective comprised of a heterogenous group of patients, the characteristics of which are shown in Table 1. All 17 patients were under a regular therapy with cysteamine since they were diagnosed with nephropathic cystinosis in early childhood. In order to prevent any interferences with previous doses of cysteamine, the patients were asked to take their last regular dose of cysteamine on the evening before the day of measurement.

**Table 1:** Patient characteristics

Number of patients	17
Age (yr)	22.8 ± 10.5
Children (between 2 and < / = 12)	3
Adolescents (between > 12 and < / = 21)	6
Adults (> 21)	8
Male	13
Weight (kg)	55.3 ± 21.1
Single dose of IR-Cysteamine (Cystagon®)	550.7 ± 159.6
Single dose of DR-Cysteamine (Procysbi®)	741.2 ± 203.5

The measurements were performed over a course of two days: On the first day, a single dose of immediate release cysteamine was administered to each of the 17 patients. On the second day each of the 17 patients received a single dose of delayed release cystine. Cystine and cysteamine levels were measured on both days.

Clinical Interviews were conducted during the measurement period in order to assess the onset of adverse effects.

### Blood collection scheme

At each measurement point, 10 ml of EDTA-blood were collected for the determination of cystine and 1 ml of heparinized blood for the determination of cysteamine.

Under IR-cysteamine, blood samples for the measurement of cysteamine were taken before drug intake and 30, 60, 90, 120, 180, 240, 300 and 360 min thereafter. Blood samples for the measurement of cystine were taken before drug intake and after 90, 180 and 360 min.

For DR-cysteamine, the blood samples were taken in the same manner, but with additional blood samples after 540 and 720 min due to the prolonged dosing interval.

### Dosage of IR-cysteamine (Cystagon®) and DR-cysteamine (Procysbi®)

Cystagon® was dosed according to its prescribing information: Up to the age of 12 years, the dosage is calculated based on the patients’ body surface (1.30 g/m^2^ per day, divided into 4 doses). Patients who are older than 12 years or weigh more than 50 kg receive 2 g per day, divided into 4 single doses.

According to its prescribing information, Procysbi® should also be dosed according to patients’ body surface. (1.30 g/m^2^ per day, divided into 2 doses).

The dosage of Procysbi® was lowered to 70% of the equivalent dose of Cystagon® for 12 h as suggested by Langman et al. [14] who performed a randomized clinical trial comparing the effectiveness of Cystagon® and Procysbi®.

### Measurement of cystine levels

The analysis of cystine levels was performed in the laboratory of metabolic diseases, Department of General Pediatrics, University Hospital Muenster.

After isolation of the leucocytes from blood samples by sedimentation, centrifugation and repeated washing steps, N-Ethylmaleimid (NEM) was added as an alkylating agent. To deproteinize the sample, sulfosalicylacid was added. The amino acid norvaline was used as an internal standard.

Protein concentration was measured according to the method of Lowry et al. [15].

Based on the principles described by Spackman, Moore and Stein [16], column chromatography was used to analyse cystine levels.

While cystine values can also be expressed as nmol hemicystine/mg protein, the unit of nmol cystine/mg protein was used here.

WBC cystine levels under 0.5 nmol cystine/mg protein (= 1 nmol hemicystine/mg protein) are considered as the therapeutic goal [17].

### Measurement of cysteamine levels

The measurement of cysteamine took place at the metabolic laboratory of the Hospital Necker Enfants Malades, University of Paris. The determination of cysteamine levels was performed via liquid chromatography–MS/MS [18]. Cysteamine levels are referred to in µmol/l.

### Statistical analysis

Mean values, standard deviation ranges, C_max_ and T_max_ were calculated with SPSS. As data were not distributed normally, statistical significance was tested with the Wilcoxon test with SPSS. A p-value lower than 0.05 was considered statistically significant. Boxplots and Areas under the curve were calculated with Microsoft Excel for Mac.

## Limitations

Due to an error in the collection of blood samples of one of the 17 patients, cysteamine levels could not be measured in one case.

### Consent of local ethic board

Data analysis was consented by the local ethics board (number 2019-199-f-S).

## Results

### Patients characteristics

Data of 17 patients with a mean age of 22.9 years were analysed retrospectively. The dosage of delayed-release cysteamine was 70% of the equivalent 12 h dose of immediate-release cysteamine. Further patient characteristics are summarized in Table 1.

**Fig. 1:**
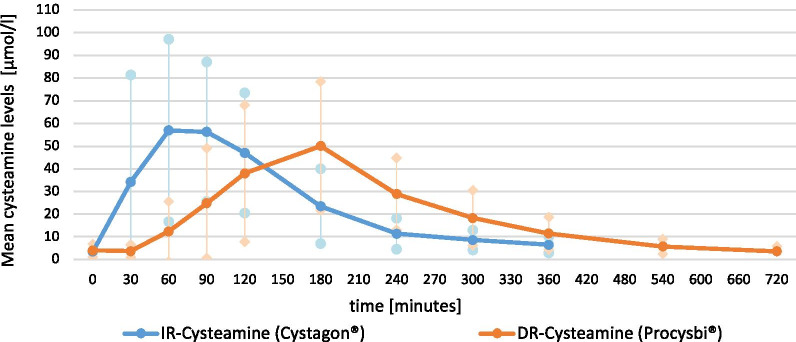
Cysteamine levels. This figure shows mean cysteamine levels in µmol/l under a single dose of IR-cysteamine (Cystagon®, blue) and DR-cysteamine (Procysbi®,orange) over a period of 360 min, 720 min respectively. Maximum cysteamine levels are reached after 60 min under Cystagon® and after 180 min under Procysbi®. The difference in the maximum cysteamine concentrations was not statistically significant (p = 0.28)

Cysteamine levels were compared under IR-cysteamine (Cystagon®) and DR-cysteamine (Procysbi®) at different time-points, which are displayed in the curves in Fig. 1.

There was no difference in the patients’ initial cysteamine levels at the beginning of the measurement. While the mean peak cysteamine level under IR-cysteamine was reached after a mean time of 60 min, the mean peak cysteamine level under DR-cysteamine was reached after 180 min, as can be seen in Fig. 1. Thus, cysteamine concentration rose more rapidly under IR-cysteamine than under DR-cysteamine. The difference in the peak cysteamine concentrations after 60 min with IR- cysteamine and after 180 min with DR- cysteamine was not statistically significant (p = 0.57).

The differences in cysteamine values at the other measurement points—except at the beginning and after 120 min—are statistically significant, as shown in Table 2.

**Table 2:** Mean cysteamine levels

Time in minutes	Mean Cysteamine level (Cystagon®)	Mean Cysteamine level (Procysbi®)	p-value
0	3.49 ± 1.8	4.04 ± 2.88	0.96
30	34.23 ± 47.22	3.72 ± 2.78	0.001
60	56.95 ± 40.21	12.39 ± 13.16	0.002
90	56.33 ± 30.8	24.81 ± 24.24	0.002
120	47.01 ± 26.53	37.98 ± 30.09	0.43
180	23.5 ± 16.44	50.03 ± 28.46	0.002
240	11.4 ± 6.79	28.9 ± 15.87	0.001
300	8.63 ± 4.38	18.33 ± 12.19	0.003
360	6.53 ± 3.62	11.52 ± 7.08	0.016
540		5.77 ± 3.26	
720		3.61 ± 2.23	

This table demonstrates mean cysteamine levels in µmol/l under a single dose of IR-cysteamine (Cystagon®) and DR-cysteamine (Procysbi®) at different point of times, including standard deviations and p-values

We also compared the mean maximum serum concentration of cysteamine under IR- and DR-cysteamine (C_max_) as well as the mean time until the maximum serum concentrations were reached (T_max_). The mean maximum cysteamine levels (C_max_) under IR- cysteamine were 72.58 ± 46.45 µmol/l and 56.2 ± 28.3 µmol/l with DR-cysteamine (p = 0.28), as shown in Table 3. The T_max_ is more than two folds higher under DR-cysteamine than under IR-cysteamine, which underlines the delay in the rise of cysteamine concentration, as already described in Fig. 1.

**Table 3:** Pharmokinetic parameters

Parameter	IR-cysteamine (Cystagon®)	DR-cysteamine (Procysbi®)	p-value
Mean minimum concentration of cystine (in nmol cystine/mg protein)	0.38 ± 0.3	0.41 ± 0.41	0.59
Mean C_max_ of cysteamine (in µmol/l)	72.58 ± 46.45	56.2 ± 28.3	0.28
Mean T_max_ of cysteamine (in minutes)	75.0 ± 24.5	159.38 ± 39.07	0.001
Time with cystine values under 0.5 (in minutes)	196.46	216.71	0.55
AUC cysteamine (after 1 dosing)	9400.8	11,578.8	
AUC cysteamine (24 h)	37,603.2	23,157.6	

This table shows mean values incl. standard deviations and p-values of pharmacokinetic parameters of 17 patients after ingestion of a single dose of IR-cysteamine (Cystagon®), DR-cysteamine (Procysbi®) respectively

The AUC for IR- and DR-cysteamine after a single dose (6 h and 12 h respectively) were calculated based on our data. In order to better compare the AUC, they were extrapolated to a 24 h interval. The AUC for IR-cysteamine was 37,603.2 and for DR-cysteamine 23,157.6.

The exact data of the cysteamine values, including standard deviation and p-values at each measurement point as well as the AUC for both formulas are displayed in Tables 2 and 3 respectively.

**Fig. 2:**
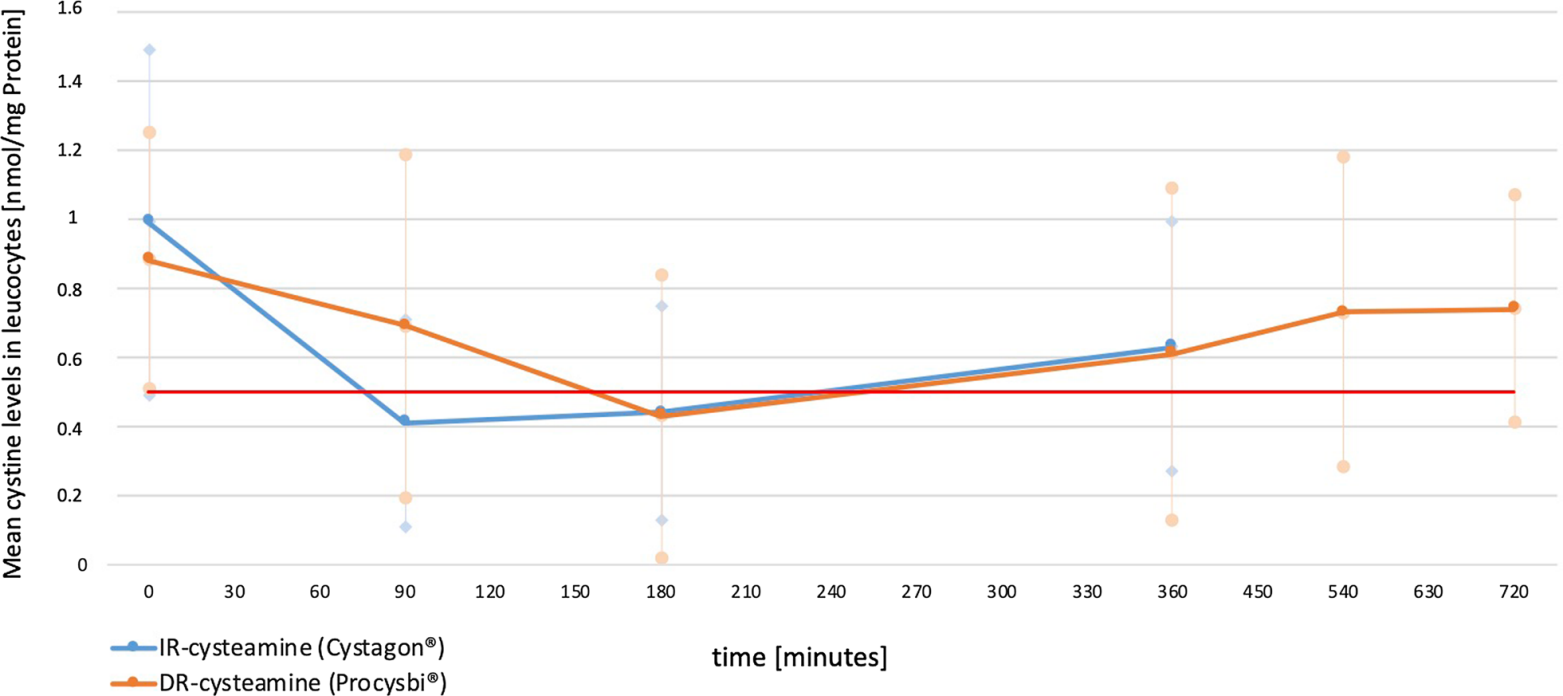
Mean cystine levels. This figure shows the mean cystine levels of all patients, including standard deviations, when taking IR-cysteamine (Cystagon®, blue) as well as DR-cysteamine (Procysbi®, orange) over a period of 360 min, 720 min respectively. Initially, patients’ cystine levels are similar under both formulas. Mean cystine levels reach their trough value after 90 min under IR-cysteamine and after 180 min under DR-cysteamine. Both drugs lead to the target value of cystine under 0.5 nmol cystine/mg protein

We compared the mean cystine levels under IR-cysteamine and DR-cysteamine at different points of measurement (Fig. 2). The corresponding data incl. standard deviations and p-values are demonstrated in Table 4.

**Table 4:** Mean cystine levels

Time in minutes	Cystine level (Cystagon®)	Cystine level (Procysbi®)	p-value
0	0.99 ± 0.5	0.88 ± 0.37	0.55
90	0.41 ± 0.3	0.69 ± 0.5	0.007
180	0.44 ± 0.31	0.43 ± 0.41	0.45
360	0.63 ± 0.36	0.61 ± 0.48	0.8
540	–	0.73 ± 0.45	–
720	–	0.74 ± 0.33	–

This table demonstrates mean cystine levels (in nmol cystine/mg protein) under a single dose of Cystagon® and Procysbi® at different point of times, including standard deviations and p-values

At the initial measurement point, there was no difference in the patients’ mean cystine levels. The mean minimum concentration of cystine was reached after 90 min with IR-cysteamine (Cystagon®) and after 180 min with DR-cysteamine (Procysbi®). The mean minimum cystine concentrations were 0.38 ± 0.3 nmol cystine/mg protein with IR-cysteamine and 0.41 ± 0.41 nmol cystine/mg protein with DR-cysteamine (p = 0.59, Table 3).

The mean times until the mean minimum cystine concentrations were reached were 116.47 ± 42.27 min for IR-cysteamine vs 201.18 ± 81.3 min for DR-cysteamine (p = 0.001).

Cystine levels rose to a mean maximum concentration of 0.63 ± 0.36 nmol cystine/mg protein after 360 min under IR-cysteamine. With DR-cysteamine, after 360 min, the mean cystine concentration was 0.61 ± 0.48 nmol cystine/mg protein. There was no statistical difference between the cystine concentrations with both drugs after 360 min. Between 180 and 360 min, cystine levels were similar.

After 540 and 720 min, data for cystine exists for DR-cysteamine only, showing an increase in cystine levels.

Both drugs reach the recommended target values under 0.5 nmol cystine/mg protein. We calculated the duration that patients had cystine values under 0.5 nmol cystine/mg protein, which showed no statistically significant difference (196.46 ± 149.22 min (IR-cysteamine vs 216.71 ± 117.9 min (DR-cysteamine p = 0.55).

Cystine levels above the target value of 0.5 nmol cystine/mg protein were reached after a mean time of 250 min with IR-cysteamine as well as with DR-cysteamine.

At the end of each drugs’ dosing interval (360 min for IR-cysteamine, 720 min for DR-cysteamine) mean cystine values were 0.63 ± 0.36 nmol cystine/mg protein vs 0.74 ± 0.33 nmol cystine/mg protein. (p = 0.05).

### Side effects

88.2% of the patients (15/17) reported gastrointestinal side effects, such as nausea, vomiting and/or abdominal pain as well as a sulfurous body odor after administration of IR-cysteamine. With DR-cysteamine, only 6 patients (35.3%) suffered from gastrointestinal side effects, of which 4 patients described them as being less severe than with IR-cysteamine. 5 patients perceived sulfurous body odor, of which 3 patients likewise described this side effect as being less intense. No other acute side effects were reported.

## Discussion

Based on experimental findings that the C_max_ and AUC of cysteamine as well as its effect on decreasing cystine levels was greater from the small intestine than from the stomach [19, 20], a delayed-release formula of cysteamine bitartrate (Procysbi®, Horizon Pharma USA and Chiesi Farmaceutici S.p.A., Parma, Italy) was developed. To achieve this, cysteamine is encapsulated in gastric-acid resistant beads. Thus, the drug is released in the small intestine instead of the stomach, as it is the case with immediated release cysteamine.

As described above, our data confirmed the delayed release of DR-cysteamine (Procysbi®), as the maximum effect on cystine was delayed by approximately 90 min compared to IR-cysteamine (Cystagon®). With DR-cysteamine, the C_max_ was reached approximately 120 min later than with IR-cysteamine. Additionally, the T_max_ for DR-cysteamine was more than two folds higher than for IR-cysteamine, underlining its release in the small intestine. These findings are in line with those of a previous analysis of Langman et al. [14].

Likewise, we could confirm the findings of Belldina et al. [21] who saw a lag time (mean lag time 0.44 h) between the drug concentration of immediate-release cysteamine and its effect: Our data showed that while the C_max_ is reached already after 60 min, the mean minimum cystine concentration is reached after 90 min.

Our data showed no statistically significant difference in the effectiveness of IR-cysteamine and DR-cysteamine in depleting white blood cell cystine levels. Cystine levels were effectively decreased under 0.5 nmol cystine/mg protein under both drugs. There was an average decrease in cystine levels of 58.59% under IR-cysteamine and 51.14% under DR-cysteamine. In terms of its effect on decreasing mean absolute WBC cystine content, we can confirm previous findings of Langman et al. who showed a non-inferiority of DR-cysteamine in comparison to IR-cysteamine [14]. Dohil et al. compared a self-manufactured formula of enteric-coated cysteamine with cysteamine and likewise showed that both formulas effectively decrease WBC cystine levels [22].

It is not only of interest how much cystine levels are decreased, but also how long WBC cystine levels are kept under the target value of 0.5 nmol cystine/mg protein. It has already been proven that immediate release cysteamine is able to keep WBC cystine under the target level of 0.5 nmol/mg protein throughout the whole dosing interval [21]. Our results showed that there was no statistically significant difference in how long the target levels were kept and the point of time when the target value was crossed: under IR-cysteamine, cystine levels rose above 0.5 nmol cystine/mg protein after a mean time of 250 min, indicating a slight underdosing as the next dose is not scheduled until 110 min later. Under DR-cysteamine, cystine levels above the target values were also reached already after 250 min: As the next dosing interval is scheduled for almost 8 h later, we suggest that the dosing was probably insufficient, otherwise cystine levels would reach the target value, but would stay there for an insufficient amount of time. The probable underdosing is also suggested by the fact, that at the end of the dosing interval of DR-cysteamine, the mean cystine value had already risen to mean cystine values of 0.74 ± 0.33 nmol cystine/mg protein. With the dose being used in our study, DR-cysteamine would likewise have to be administered more frequently.

Our data do not support the results of Langman et al. [14] and Dohil et al. [22] who considered 70–80% and 60% respectively of delayed-release cysteamine of the total daily dose of IR-cysteamine dosing as being sufficient. We therefore suggest to initiate therapy with a higher dosing than 70% of the previous cysteamine dosing for the treatment of nephropathic cystinosis, according to the prescribing information (1.30 g/m^2^ per day, divided into 2 doses). The dosing should then be adjusted according to the results of regular measurements of the cystine levels.

Adherence to cysteamine therapy is challenging, mainly due to the frequent dosing scheme which interrupts patients’ sleep on a daily basis but also because of the numerous side effects, such as nausea and body odor. These side effects have serious implications on patients social life and therefore lead to a diminished compliance, especially in adolescent patients [11, 12].

In terms of side effects, our study showed a clear advantage for the usage of DR-cysteamine, as the number of patients complaining about nausea and/or body odor or halitosis was much lower, despite the fact that the single dose of DR-cysteamine was higher than a single dose of IR-cysteamine. This effect can be attributed to the release of DR-cysteamine in the small-intestine instead of the stomach, due to its enteric coating, which directly contributes to less gastrointestinal side effects. These findings confirm those of other studies, who also reported ameliorated or even no side effects under delayed-release cysteamine [23], but are opposed to those of Langman et al. who reported more side effects under DR-cysteamine [14].

The fact that Procysbi® needs to be administered only twice daily could therefore be yet another reason for an increase in patients’ compliance as the sleep is not interrupted. Our data suggest that a dosing interval of 12 h would result in an underdosing when giving only 70% of the IR-cysteamine dosing. As previously stated, to achieve satisfactory cystine levels under the target value of 0.5 nmol cystine/mg protein, the dose of DR-cysteamine should probably be increased. This fact is shown by the calculated AUC for IR—and DR cysteamine for 24 h, which is more than 1.5 fold as high with IR-cysteamine than with DR-cysteamine. Still, more frequent intakes might be necessary as drug release is mainly delayed and not significantly retarded. As seen in Fig. 2, cystine levels increase above the target value of 0.5 nmol cystine/mg protein before the next dosing is scheduled. This indicates that a 12 h dosing interval as approved by the manufacturer is insufficient. Our data suggests to increase the intake of Procysbi® to three times daily, which is already common practice recommended by some pediatricians. Thereby, a steadier decline in cystine levels might be achieved while patients still benefit from a more convenient dosing scheme. Data on how cystine levels are decreased by this dosing regimen need to be studied further.

Strict adherence to therapy with IR-cysteamine significantly delays the onset of complications, such as ESRD, hypothyroidism and diabetes and thus improves patients life expectancy, provided that therapy is started as early as possible [9]. These benefits can only be reached by a diligent patients’ compliance, which we believe can be significantly ameliorated by taking DR-cysteamine, due to its fewer side effects and more comfortable dosing scheme provided that doses are increased.

Nevertheless, as performed in our department, it should be reviewed individually if IR-cysteamine or DR-cysteamine is better tolerated by patients, as there are single cases of patients who report even more side effects under DR-cysteamine [24].

It is important to investigate the long-term effects of DR-cysteamine. As described above, numerous data about the therapeutic benefits of immediate-release already exist. In the setting of a follow-up, Langman et al. have proven optimal cystine levels of 40 patients that had been treated with delayed-release cysteamine for a period of 2 years while additionally the patients’ quality of life was improved [25]. These findings are confirmed by Dohil et al., who did a 6 year follow up of 2 patients treated with the self-manufactured formula of delayed-release cysteamine and likewise concluded satisfactory cystine levels [26].

Nevertheless, Bäumner et al. reported two patients with decreased kidney function under treatment with delayed-release cysteamine for a period of 9 months [24].

Further studies are needed to obtain more data regarding the long-term effects of delayed-release cysteamine on cystine levels and kidney function.

Regarding the limitations of our evaluation, it must be taken into consideration that the data came from a small number of patients (n = 17) which minimizes statistical significance. However, as nephropathic cystinosis is a rare disease with an estimated number of 150 patients in Germany, it is difficult to obtain data from a higher number of patients. Nevertheless, it is important to analyze these data even when their number is limited, to gain more knowledge regarding this rare disease.

It must also be taken into consideration that most of the patients in our study had very well controlled cystine levels. However, insufficiently controlled cystine levels due to a lack of compliance are a common problem in patients with nephropathic cystinosis [27]. We do not have any data on how cystine levels would decrease in patients with higher baseline levels of cystine. Although the pharmacokinetic profile of the administered drugs can be assumed to remain unchanged, the effect on cystine levels might differ in these patients. Higher doses might be required to achieve a sufficient reduction in cystine levels. Further data need to be obtained to validate this hypothesis.

Furthermore, data were obtained after ingestion of a single dose of IR-cysteamine respectively DR-cysteamine. Thus, conclusions drawn from these data are limited, as they do not completely reflect reality, in which a pharmacokinetic steady state of the drugs develops over a certain period of time. These steady-state data still need to be examined in further studies.

Regarding the report of adverse side effects, it must also be taken into consideration, that patients were not blinded, which might have affected their reports.

To conclude, our findings suggest the following recommendations:

Treatment should be initiated with DR-cysteamine in patients with nephropathic cystinosis. The initial dose should be started at a higher dose than suggested by Langman et al. [14], while being individually adjusted to the patients’ regularly obtained cystine levels. Additionally, the dosing interval should be increased to 3 times per day. These measures are likely to improve drug efficacy and therapy adherence, thus leading to favorable long term outcomes.

## Data Availability

The datasets generated and analysed in this study are available from the corresponding author on reasonable request.
